# Does creatine supplementation improve the plasma lipid profile in healthy male subjects undergoing aerobic training?

**DOI:** 10.1186/1550-2783-5-16

**Published:** 2008-10-03

**Authors:** Bruno Gualano, Carlos Ugrinowitsch, Guilherme G Artioli, Fabiana B Benatti, Fernanda B Scagliusi, Roger C Harris, Antonio H Lancha

**Affiliations:** 1 Laboratory of Applied Nutrition and Metabolism, School of Physical Education and Sport, University of São Paulo, Brazil; 2 Laboratory of Rheumatology Assessment and Conditioning, Division of Rheumatology, School of Medicine, University of São Paulo, Brazil; 3 School of Sport, Exercise and Health Sciences, University of Chichester, UK

## Abstract

We aimed to investigate the effects of creatine (Cr) supplementation on the plasma lipid profile in sedentary male subjects undergoing aerobic training.

Subjects (n = 22) were randomly divided into two groups and were allocated to receive treatment with either creatine monohydrate (CR) (~20 g·day^-1 ^for one week followed by ~10 g·day^-1 ^for a further eleven weeks) or placebo (PL) (dextrose) in a double blind fashion. All subjects undertook moderate intensity aerobic training during three 40-minute sessions per week, over 3 months. High-density lipoprotein cholesterol (HDL), low-density lipoprotein cholesterol (LDL), very low-density lipoprotein cholesterol (VLDL), total cholesterol (TC), triglyceride (TAG), fasting insulin and fasting glycemia were analyzed in plasma. Thereafter, the homeostasis model assessment (HOMA) was calculated. Tests were performed at baseline (Pre) and after four (Post 4), eight (Post 8) and twelve (Post 12) weeks.

We observed main time effects in both groups for HDL (Post 4 versus Post 8; *P *= 0.01), TAG and VLDL (Pre versus Post 4 and Post 8; *P *= 0.02 and *P *= 0.01, respectively). However, no between group differences were noted in HDL, LDL, CT, VLDL and TAG. Additionally, fasting insulin, fasting glycemia and HOMA did not change significantly.

These findings suggest that Cr supplementation does not exert any additional effect on the improvement in the plasma lipid profile than aerobic training alone.

## Introduction

Several recent studies have indicated that creatine (Cr) supplementation may reduce the symptoms and progression of certain diseases, including mitochondrial cytopathies [1], neurodegenerative diseases [2] and glucose intolerance [3]. In addition there is evidence that Cr supplementation may also improve the lipid profile when combined with exercise training. Kreider et al. [4] reported higher plasma HDL-cholesterol levels and lower VLDL-cholesterol in Cr-supplemented athletes undergoing resistance and agility-sprint training compared to their non-supplemented counterparts, while Arciero et al. [5] reported lowered plasma total cholesterol concentrations after 28 days of Cr supplementation combined with resistance training. In contrast, Kreider et al [6] did not observe any changes in the plasma lipid profile of athletes after 21 months of Cr supplementation. However, none of the aforementioned studies were undertaken with the specific intention of examining the changes in plasma lipid profile. The designs of these studies did not allow any conclusion regarding possible additive effects of Cr supplementation on the plasma lipid profile during training.

To our knowledge, only Earnest et al. [7] reported a specific effect of Cr supplementation on the plasma lipid profile. This group observed that physically active subjects with high basal cholesterol levels exhibited a reduction in blood total cholesterol, triglycerides and VLDL-cholesterol, after 12 weeks of Cr supplementation. The authors attributed these changes to a reduction in blood glucose (*P *= 0.051) brought about by Cr supplementation. Reduced blood glucose was interpreted to indicate an enhancement in insulin sensitivity, which simultaneously could improve the lipid profile if followed by a reduction in fasting insulin levels [7]. It is known from other studies that, changes in glycemia and/or insulinemia may directly affect blood lipoproteins [8-10].

Pedersen and Saltin [11] have reported that aerobic training has a positive effect on blood lipoproteins. Independently, Walsh et al. [12] have indicated *in vitro *a regulatory effect of creatine (Cr) and phosphorylcreatine (PCr) on oxidative phosphorylation in mitochondria which is consistent with a model first proposed by Wyss et al [13], as well as the boader view that the primary role of the Cr-PCr/CK system is the regulation of ADP homeostasis [14]. These authors demonstrated that PCr decreases the sensitivity of mitochondrial respiration to ADP whereas Cr has the opposite effect [12]. Several investigations have shown increases in Cr content with little or no change in PCr following Cr supplementation [15,16]. This would lead to a reduced PCr/Cr ratio, favouring an increase in intra-mitochondrial [free ADP]. Walsh et al. [12] noted that a combination of increases in [free ADP] and [Cr] at rest would elevate mitochondrial respiration and oxidative phosphorylation after Cr supplementation. This raises the possibility that Cr supplementation could exert an additive effect to aerobic training on the balance of lipid and carbohydrate utilisation as fuels during training, with long term effects on the blood lipoprotein profile. Theoretically this could provide an explanation for the positive effects observed by Kreider et al. [4], Arciero et al. [5] and Earnest et al. [7] (though we note again that no similar effect was observed by Kreider et al [6]).

We have recently demonstrated that Cr supplementation and aerobic training have an additive effect on glucose tolerance, which could not be attributed to an enhanced training capacity alone [3]. From this we hypothesized that the inclusion of Cr during aerobic training may also affect the plasma lipid profile, particularly in early training. The aim of this study was therefore to test the possible synergistic effects of Cr supplementation and aerobic training on the plasma lipid profile, glucose and insulin levels in healthy, sedentary, males.

## Materials and methods

### Subjects

Participants were recruited between August and December 2005 from Sao Paulo, Brazil, through advertisement in local newspaper and distribution of leaflets to homes. Respondents were invited to take part in the study if they were sedentary males (18 – 35 years old) for at least the past seven years, without pre-existing renal or cardiovascular diseases, eutrophic, non-smoker, and non-vegetarian. Subject details are given in table 1.

**Table 1:** Subject characteristics in CR (n = 12) and PL (n = 10) groups.

	**CR group**	**PL group**
Age (years)	24.4 ± 5.0	24.2 ± 4.2
BMI (kg·m^-2^)	23.2 ± 4.6	24.1 ± 3.8
Body Fat Content (%)	17.3 ± 4.1	17.6 ± 3.8
Waist-to-hip ratio (cm)	0.84 ± 0.06	0.83 ± 0.08
Waist circumference (cm)	81.1 ± 8.8	78.3 ± 10
VO_2max _(ml·kg^-1^·min^-1^)	34.6 ± 6.6	35.3 ± 4.3
VT (ml·kg^-1^·min^-1^)	32.7 ± 6.2	28.9 ± 5.9
Resting HR (beats·min^-1^)	66.0 ± 6.2	65.8 ± 7.5

No significant differences were observed between groups (unpaired t test). VO_2peak _= peak oxygen consumption; VT = Ventilatory Gas Exchange Threshold; HR = Heart Rate

The study was approved by the University's ethics committee and all subjects signed an informed consent form before participation.

### Experimental Protocol

A 12-wk, double-blind, randomized, placebo-controlled trial was conducted between 1 March and 1 June 2006. Subjects were randomly assigned to receive either Cr (CR; n = 12) or placebo (PL; n = 10). Both groups were submitted to moderate intensity aerobic training for three months. Blood draws were performed prior to training (Pre), after 4 (Post 4), 8 (Post 8) and 12 weeks (Post 12). Samples were centrifuged at 4000 rpm, 4°C, for 5 minutes and stored in a -70°C freezer. We analyzed total cholesterol, triglycerides, HDL-cholesterol, LDL-cholesterol and VLDL-cholesterol. Fasting plasma insulin and fasting plasma glucose were also determined and used to estimate *homeostasis model assessment *(HOMA) *index *[17]. All analyses were performed in duplicate and the mean value was calculated.

Possible differences between groups and changes during treatment in dietary intake were assessed by three-day 24-hour dietary recall at baseline and Post 12.

### Cr supplementation and blinding

The CR group received 0.3 g (creatine monohydrate)·day^-1^·kg ^-1 ^of body weight during the first week (loading phase) and 0.15 g·day^-1^·kg ^-1 ^of body weight for the next 11 weeks (maintenance phase); the PL group was given dextrose in place of Cr, at the same dose. Body weights were those measured at the start of the study. This resulted in absolute doses in high body weight subjects slightly higher that those usually used for loading and maintenance (see also Discussion). During the loading phase supplements were presented in four packages, with equal content, and subjects were instructed to ingest one supplement package at breakfast, lunch and dinner and the fourth at 10 pm. During the maintenance phase the dose was divided into two with subjects consuming the supplement during lunch and dinner. The packages were coded so that neither the investigators nor the participants were aware of the contents until completion of the analyses. All subjects were instructed to dissolve the supplements, preferably in juice, in order to mask both the low solubility of Cr and the taste of dextrose. To evaluate subject blinding, the questionnaire asked subjects to indicate which treatment they believed they have received (Cr or placebo) at end of study. The compliance to Cr supplementation was monitored weekly by personal communication. The subjects completed a weekly questionnaire regarding possible adverse effects of Cr supplementation (adapted from Volek et al. [18]).

In order to verify the purity of the Cr used, a sample was analysed by HPLC [19] and 99.9% purity was established.

### VO_2peak _test

VO_2peak _was determined using an incremental treadmill exercise test conducted according to the Bruce protocol. This test is conducted in three minutes stages. The Bruce protocol begins with a speed of 2.74 km·hour ^-1 ^and a slope of 10% corresponding to a work rate of approximately 4.6 METS. Each 3 minutes the workload is increased by a combination of increasing the speed and the grade of the treadmill. Attainment of VO_2max _was accepted when two of three criteria were met: a plateau in VO_2_, a respiratory exchange ratio (RER)> 1.1 and volitional exhaustion. Ventilatory gas exchange threshold (VT) was determined by the V-slope method[20].

### Exercise training

Both groups were submitted to aerobic training, three times a week, for 40 minutes per session, for 3 months. Running intensity was set at the corresponding heart rate of 70% of VO_2peak _determined by an incremental VO_2peak _test. Subjects were requested to run at the pre-set heart rate during the whole training period. Thus, an increase in both running distance and running intensity were expected as training progressed. All sessions were monitored by a fitness-professional. From the outset it was ruled that subjects would be dropped from the study if they missed three non-consecutive training sessions, or two consecutive sessions. One subject from PL group was excluded for this reason.

### Food intake assessment

Food intake was assessed by three 24-hour dietary recalls, using visual-aid photographs of real foods. Energy and macronutrient intakes were examined by the Brazilian software Virtual Nutri^®^. Additional information was obtained from the Brazilian Table of Food Composition (version 1.0, 1997, University of Sao Paulo, Sao Paulo, SP) When data from particular foods were lacking, data from a related food was used.

### Plasma analyses

#### Fasting plasma glucose

Fasting plasma glucose was determined by colorimetric enzymatic assay, with a commercial kit (Glicose Líquido Estável^®^, Bioclin, Belo Horizonte).

#### Fasting plasma insulin

Fasting plasma insulin was determined by radioimmunoassay technique (DPC^®^, Diagnostic Products Corporation, Los Angeles, CA)

#### Total cholesterol Level

Total serum cholesterol concentration was determined as previously described by Kaplan and Pedersen [21], with a colorimetric enzymatic assay (GPO/POD), and with a commercial kit aid (CELM^® ^– Colesterol, Sao Paulo).

#### HDL-cholesterol

HDL-cholesterol was determined according to Finley et al. [22] using a commercial kit (CELM^® ^– HDL-colesterol, Sao Paulo).

#### Triglycerides, VLDL-cholesterol and LDL-cholesterol

Triglycerides were assayed according to Kaplan and Pedersen [21], by colorimetric enzymatic assay (GPO/POD), using a commercial kit (CELM^® ^– Triglicerídeos, Sao Paulo). From this VLDL-cholesterol (VLDL-cholesterol = tryglicerides/5) and LDL-cholesterol (LDL-cholesterol = total cholesterol - (HDL-cholesterol + VLDL-cholesterol)) concentrations were calculated.

### Statistical analyses

SAS^® ^proc Mixed was used to analyze repeated measures and, when applicable, Tukey Post hoc was used for multiple comparisons. The groups (CR and PL) and periods (PRE, POST 4, 8 and 12 weeks) were considered fixed factors and the subjects were assigned as random factors.

For baseline comparisons between the groups two-sample independent t-test was used. All data is expressed as mean ± SD. The significance level adopted to reject the null hypothesis was p ≤ 0.05.

Also, SAS^® ^proc IML was used to determine the power of the statistical tests using a simulation model [23] as suggested by Littell et al. [24]. Expected means and variance were used as input to simulate lipid profile data using an autoregressive correlation structure within subjects measurements. Our sample produced a power ranging between 0.8 and 0.9 in the comparison between groups for total cholesterol, HDL-cholesterol, TAG and VLDL-cholesterol, the most important dependent variables.

## Results

Of the 103 people who responded to the initial request for volunteers, 79 were excluded as they performed regular physical activity or expressed self-reported chronic disease. The remaining 24 subjects were randomly assigned to the CR or PL groups. Two subjects were subsequently lost in the PL group: one withdrew for personal reasons and one was excluded because they missed 2 consecutive training sessions. Consequently 12 subjects remained in the Cr group and 10 in the PL group.

There were no differences between or within groups in both nutrient and energy intake (Table 2). The blinding was considered successful because the ability of participants to accurately guess their group assignment was no better than chance in both groups and was not different between groups (CR: 43% and PL: 45%, *P *= 0.89).

**Table 2:** Food intake by subjects in the CR (n = 12) and PL (n = 10) groups, assessed by three 24-hour dietary recall at baseline and after 12 weeks.

	**CR**		**PL**	
		
	Pre	Post 12	Pre	Post 12
**Energy (kcal)**	2927 ± 321	2891 ± 295	2687 ± 209	2715 ± 216
**Protein (%)**	29.5 ± 4.3	30.0 ± 2.3	29.3 ± 3.1	30.2 ± 3.7
**Lipid (%)**	24.9 ± 2.2	25.2 ± 4.3	25.9 ± 4.1	25.8 ± 3.1
**Carbohydrate (%)**	46.2 ± 5.8	44.3 ± 5.5	44.1 ± 5.8	43.0 ± 4.9
**Protein/body weight (g·kg^-1^)**	1.1 ± 0.2	1.2 ± 0.1	1.1 ± 0.3	1.1 ± 0.3

No significant differences were observed (unpaired t test).

Twelve weeks of aerobic training significantly increased the running distance per session (CR: 42.1 ± 6.3% and PL: 36.8 ± 7.9%, *P *= 0.05 time main effect). There was also reduction in body fat content measured by skinfold thickness (CR: -26.6 ± 3% and PL: -25.9 ± 3.9%, *P *= 0.04) and resting heart rate (CR: -9.09 ± 2.2% and PL: -8.9 ± 2.8%, *P *= 0.05).

Despite the high absolute dose of Cr ingested by subjects with the highest body weights (compared with ACSM recommendations in 2000 [25]), there were no reports of any deleterious effects assessed through the subjective questionnaire.

**Figure 1:**
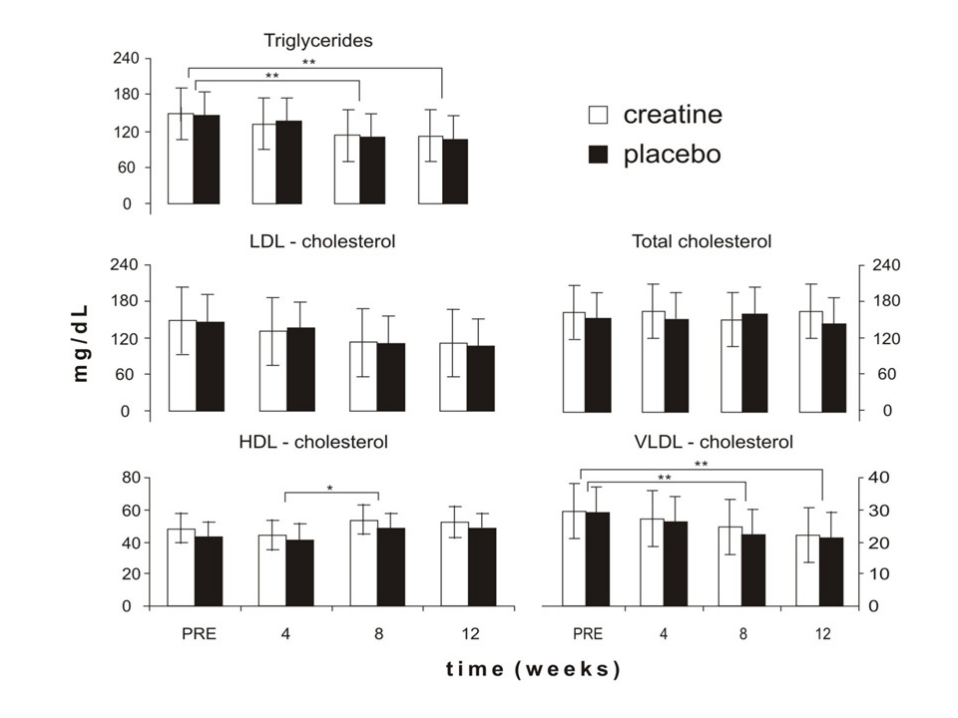
**Effects of Cr supplementation in conjunction with aerobic training on blood triglycerides, VLDL-cholesterol, total cholesterol, LDL-cholesterol and HDL-cholesterol at baseline (Pre) and after 4 (Post 4), 8 (Post 8), and 12 (Post 12) weeks of intervention.** * main time effect: Post 4 versus Post 8 (p = 0.01); * * main time effect: Pre versus Post 8 and Post 12 (p = 0.02 and p = 0.01, respectively). No significant interaction effects were observed between the CR (n = 12) and PL (n = 10) groups.

Lipid profile values can be found in Figure 1. There was no significant difference between groups at baseline. However, there were increases in HDL-cholesterol (Post 4 versus Post 8; *P *= 0.01) and decreases in TAG and VLDL-cholesterol (Pre versus Post 4 and Post 8; *P *= 0.02 and *P *= 0.01, respectively) in both groups (main time effect). No variable was significantly changed with Cr supplementation (interaction effect).

No significant main effects or differences between groups were found in fasting glucose, fasting insulin and HOMA index results (Table 3).

**Table 3:** Fasting glucose (mg/dl), insulin (μUl/ml) and Homa index.

	**PRE**	**POST 4**	**POST 8**	**POST 12**
***Fasting glucose***				
CR	77,7 ± 11,7	70,4 ± 8,7	67,2 ± 11,4	68,8 ± 17,3
PL	77 ± 10,8	72 ± 6,7	65 ± 12,4	76,4 ± 12,4
***Fasting insulin***				
CR	11.96 ± 5.95	13.89 ± 7.39	15.66 ± 4.54	15.32 ± 6.17
PL	13.54 ± 6.87	12.57 ± 3.76	14.43 ± 4.77	14.59 ± 8.32
***HOMA***				
CR	2.47 ± 1.36	2.68 ± 0.61	2.71 ± 1.06	2.13 ± 1.07
PL	2.14 ± 0.59	2.11 ± 0.64	2.48 ± 0.90	2.58 ± 1.53

Fasting glucose, fasting insulin and Homa index for the CR (n = 12) and PL (n = 10) groups at Pre and 4, 8 and 12 week-post periods. The analysis of variance did not show significant differences.

## Discussion

As body weights in the CR group ranged from 64.3 to 110.0 kg, absolute doses of Cr given in the first week ranged from 19.3 to 33.0 g·day^-1^, with 50% less during the following eleven weeks. The mean body weight of the CR group was 83.1 ± 16.3 kg. The dose of 0.3 g·day^-1^·kg ^-1 ^was chosen to ensure that subjects even at the lowest body weights received a loading dose close to 20 g per day, although the same relative dose resulted in a higher loading dose in subjects at the upper end of the weight range. However, we were cognisant that some authors have suggested that use of a low Cr dose may have been responsible for the lack of change in lipid profile in the study of Volek et al. [18]. The doses of Cr used in this study are comparable to the fixed dose of 20 g·day^-1 ^used by Arciero et al. [5] during the first 5 days, and 10 g·day^-1 ^over the next 3 weeks.

Following the earlier work by Earnest et al. [7] our aim was to test if Cr supplementation combined with aerobic training was an effective strategy for the improvement in the lipid profile in healthy sedentary males. The main findings were a reduction in triglycerides and VLDL-cholesterol, and an increase in HDL-cholesterol, after moderate intensity aerobic training (main time effect). However, Cr supplementation had no further effect on lipid profile improvement. It is important to note that we did not test the effect of aerobic training *per se *on lipid profile. We tested for a possible additive effect of Cr supplementation on the previously described improvement in lipid profile after aerobic training [26,27].

Our findings do not agree with those of Earnest et al. [7]. The differences between studies may have arisen from differences in their research design. Earnest et al. [7] selected hypercholesterolemic men and women (those with total cholesterol levels higher than 200 mg/dl), while we investigated healthy sedentary males. Thus, it was expected from the outset that a lower adaptative response would be shown by our subjects since their baseline values were within the normal range (see figure 1, baseline data). In addition, Earnest et al. [7] stated that Cr supplementation reduced triglycerides and VLDL-cholesterol levels at week four, and maintained them up to week 12, compared to baseline values (-23%, -22% and -26%, after 4, 8 and 12 weeks, respectively). However, the Cr and placebo groups had different baseline values. Thus, this difference impairs the comparison between groups as is not possible to distinguish between a true treatment effect from a distinct response due to a baseline difference.

Earnest et al. [7] also reported a decreased fasting glucose trend (*P *= 0.051). According to the authors, such a decrease indicated a possible improvement in insulin sensitivity, which would cause a better lipid profile particularly if followed by a reduction in the fasting insulin levels. However, our results and others [28,29] have shown neither a significant improvement in insulin sensitivity nor a decrease in fasting glucose, after Cr supplementation. In light of these findings, we may suggest two explanations: 1) Cr supplementation has no effect on insulin sensitivity, or 2) Cr supplementation does improve insulin sensitivity slightly, but not enough to enhance the lipid profile in healthy subjects. As we [3] and others [28,30] have demonstrated that Cr supplementation may increase both glucose tolerance and glycogen content, we consider the latter hypothesis more plausible. Ferrante et al. [2] indicated that Cr intake can ameliorate hyperglycaemia, typical of transgenic Huntington disease mice, delaying the onset of diabetes. Supporting these findings, Eijnde et al. [31] verified that Cr ingestion can reduce the insulinogenic index in an animal model of inherited type 2 diabetes. Taken together, these results suggest that Cr might exert beneficial effects on glucose uptake mainly in insulin resistant conditions, although the exact mechanisms underlying these adaptations have yet to be clarified. Interestingly, Eijnde et al. [30] showed that Cr intake increases GLUT-4 protein content in line with glycogen accumulation during muscle disuse and a subsequent period of training, suggesting a role of GLUT-4 in Cr-induced glucose uptake enhancement.

Kreider et al. [4] also reported an improvement in the lipid profile of professional football players following Cr supplementation (15.75 g·day ^-1^) during 28 days of high intensity training. The supplemented group presented a significant increase in HDL-cholesterol (13%), and a significant decrease in both triglycerides and VLDL-cholesterol (-13%), compared to the placebo group, showing an additive effect of Cr supplementation. Accordingly, Arciero et al. [5] showed that intense strength training in conjunction with Cr supplementation (25 g·day ^-1 ^for 7 days followed by 5 g·day^-1 ^for 11 weeks) significantly decreased total cholesterol values (-9.9%) when compared to strength training only. Their results reinforced the hypothesis that Cr supplementation had an additive effect to training effects on the lipid profile. A possible explanation for this phenomenon was that Cr supplementation enhanced training quality indirectly (i.e. by optimizing volume and intensity). In a review manuscript, Durstine et al. [31] suggested that the lipid profile improved incrementally with training volume and intensity. Therefore, as Cr supplementation allowed subjects to train at a higher intensity/volume, then the training itself may have enhanced the effect and not the supplementation *per se*.

Cr supplementation is reported to be effective mainly in the improvement of performance during sustained or intermittent high intensity exercise and not in aerobic training [25]. Whilst Cr supplementation has not been shown to increase performance in moderate intensity aerobic activity, Walsh et al. [13] have suggested a mechanistic role for Cr in the stimulation of mitochondrial oxidative phosphorylation which clearly could have impact on the relative utilization of carbohydrate and fat as fuel sources, and indirectly could affect the plasma lipid profile. Whilst an attractive hypothesis, our observations do not support any such effect of Cr supplementation.

Volek et al. [18] similarly failed to observe any effect on the lipid profile of three months Cr supplementation and periodized resistance training. Arciero et al. [5] suggested that the lack of change in lipid profile after resistance training in Volek's study might have been a consequence of the low dose of Cr used (5 g·day^-1^). However, in our study we provided ~10 g·day^-1 ^and again no difference between the placebo and the Cr group was observed. Thus, whilst exercise is clearly important in determining the plasma lipid profile, Cr itself does not appear to have any additional effect in healthy males.

## Conclusion

Cr supplementation did not have any additive effect on the plasma lipid profile of healthy sedentary males undergoing moderate intensity aerobic training for 3 months. These results may appear contradictory to those of Earnest et al. [7] although we emphasise that this earlier study was carried out on hypercholesterolemic subjects, whereas ours were normocholesterolemic. Notwithstanding the lack of benefits in this present study we would suggest that additional studies should be carried out to investigate any indirect effect of Cr supplementation on the lipid profile as a result of subjects being able to train at a higher intensity.

## Competing interests

The authors declare that they have no competing interests.

## Authors' contributions

BG is the principal investigator of the project. BG and AHLJ designed the study. BG, CU, GGA, FBB and FBS collected the data. BG, CU, GGA, FBB, FBS and RCH conducted data analysis. All authors wrote the manuscript.
